# Image Denoising Using Sparsifying Transform Learning and Weighted Singular Values Minimization

**DOI:** 10.1155/2020/8392032

**Published:** 2020-08-04

**Authors:** Yanwei Zhao, Ping Yang, Qiu Guan, Jianwei Zheng, Wanliang Wang

**Affiliations:** Zhejiang University of Technology, HangZhou 310023, China

## Abstract

In image denoising (IDN) processing, the low-rank property is usually considered as an important image prior. As a convex relaxation approximation of low rank, nuclear norm-based algorithms and their variants have attracted a significant attention. These algorithms can be collectively called image domain-based methods whose common drawback is the requirement of great number of iterations for some acceptable solution. Meanwhile, the sparsity of images in a certain transform domain has also been exploited in image denoising problems. Sparsity transform learning algorithms can achieve extremely fast computations as well as desirable performance. By taking both advantages of image domain and transform domain in a general framework, we propose a sparsifying transform learning and weighted singular values minimization method (STLWSM) for IDN problems. The proposed method can make full use of the preponderance of both domains. For solving the nonconvex cost function, we also present an efficient alternative solution for acceleration. Experimental results show that the proposed STLWSM achieves improvement both visually and quantitatively with a large margin over state-of-the-art approaches based on an alternatively single domain. It also needs much less iteration than all the image domain algorithms.

## 1. Introduction

Noise inevitably exists in images during the process of real-world scenes acquisition by reason of physical limitations, leading to image denoising (IDN) and becomes a fundamental task in image processing. The recent IDN can be categorized as data-driven and prior-driven approaches.

The data-driven methods turn to a certain deep convolution neural network, such as Universal Denoising Net (UDN) [1] and Fractional Optimal Control Net [2], for the IDN problem. These CNN models, although have achieved great success provided with sufficient training samples, may not perform well in small-scale data applications. For example, one cannot obtain the acceptable network parameters on a single corrupted image, which is the case considered in this study. The aim of the prior-driven methods for image denoising is to renovate the inferior image by certain image prior or other properties, such as local smoothness, nonlocal similarity, low-rank structure, and so forth [3–5]. More specifically, the prior-based image denoising process means to find the inherently ideal image from the degraded one by extracting few significant factors and excluding the noisy information. It is a typical ill-posed linear inverse problem, and a widely used image degradation model can be generally formulated as follows [6–9]:

(1) $$\begin{array}{c} Y=\mathrm{HX}+N, \end{array}$$

where **X** and **Y** are both matrices representing the original image and the degraded one, respectively. **H** is also a matrix denoting the noninvertible degradation operator, and ***N*** is the additive noise.

To cope with the ill-posed problem, the general image denoising problem can be formulated as follows [9, 10]:

(2) $$\begin{array}{c} \begin{array}{cc} \min & {\left\|\mathrm{HX}-Y\right\|}_{F}^{2},\\ \text{s.t.} & F\left(X\right), \end{array} \end{array}$$

where *F*(**X**) is regarded as the image prior knowledge, including local smoothness, nonlocal similarity, low-rank, and sparsity, and ‖·‖_*F*_ denotes the Frobenius norm. According to sparsity property, the degraded image **x** (**x** is the vectorization of **X**, **x** ∈ *R*^*n*^) satisfies **x**=**Dκ**+**e**, where **D** ∈ *R*^*n*×*m*^ is a synthesis overcomplete dictionary, **κ** ∈ *R*^*m*^ is the sparse coefficient, and ***e*** is an approximation term in image domain [11]. This model is called as the synthesis model, and **κ** is the supposed sparse (‖**κ**‖_0_ ≪ *m*).

To be specific, given an image ***x***, the synthesis sparse coding problem is subjected to find a sparse **κ** to minimize ‖**x** − **Dκ**‖_2_^2^. Various algorithms have been proposed [10, 12–15] to figure out this NP-hard problem. Numerous researchers have learned the synthesis dictionary and updated the nonzero coefficients simultaneously to well represent the potential high-quality image. And these methods have been demonstrated useful in image denoising. Specifically, these synthesis models typically alternate two steps: the sparse coding updating and dictionary learning. However, the practical operation of synthesis models requires some rigorous conditions, which often violate in applications.

While the synthesis model has attracted extensive attentions, the analysis model has also been catching notice recently [16, 17]. The analysis model considers that a noisy image **x** ∈ *R*^*n*^ satisfies ‖Ω**x**‖_0_ ≪ *m*, where Ω ∈ *R*^*n*×*m*^ is regarded as an analysis dictionary, since it ‘analyzes' the image **x** to a sparse form. The essence of Ω**x** defines the subspace to which the image belongs. And the underlying ideal image is formulated as **y**=**x**+*ξ*, with *ξ* representing the noise. The denoising problem is to find ***x*** by minimizing ‖**y** − **x**‖_2_^2^ subject to ‖Ω**x**‖_0_ ≪ *m*. This problem is also NP-hard and resemblant of sparse coding in the synthesis model. Approximation algorithms of learning analysis dictionary have been proposed in recent years, which similar to the synthesis case are also computationally expensive.

More recently, a generalized analysis model named the transform learning model has been proposed, which follows the intuition that images are essential sparse in certain transform domain and can be expressed as **Wx**=***μ***+**ε**, where **W** ∈ *R*^*m*×*n*^ is the transform matrix, ***μ*** ∈ *R*^*m*^ is the sparse coefficient, and *ε* is the approximation error [18]. The distinguishing feature from the synthesis and analysis models is that approximation error *ε* of the transform learning model is in transform domain and is likely to be small. Another superiority of the transform model compared to the image domain model is that the former can achieve exact and extremely fast computations.

Instead of learning synthesis or analysis dictionary, the transform learning model aims at learning the transform matrix to minimize the approximation error*ε*. After getting the learned transform **W**, the original image is recovered by **W**^†^***μ***, where **W**^†^ is the pseudoinverse of **W**. The transform learning model has earned great success in application of image denoising in both efficiency and effectiveness [18–21].

Nonetheless, a remaining drawback is that the transform model overemphasizes transform domain but ignores the primary image domain. There is always a connection between image domain and transform domain, and this can be treated as a regularization term in image denoising.

For taking full use of the advantages of both image domain and transform domain and implementing single image denoising problem, this study focuses on sparsifying transform learning and essential sparsity property of image, and proposes a novel algorithm named sparsifying transform learning and weighted singular values minimization (STLWSM). Specifically, our model simultaneously considers the sparsifying transform learning and the weighted singular values minimization of image patches.

The remainder of this paper is organized as follows. In the next section, a brief review of the transform domain and image domain for IDN is provided. In section 3, we propose our method and obtain the efficient solution.  Section 4 provides experimental results of gray images and color images. Conclusions are drawn in section 5.

## 2. Related Works

### 2.1. Transform Domain for IDN

As mentioned in the previous section, the transform model can utilize the sparsity of image in transform domain to increase efficiency. Therefore, the analytical transform models such as wavelets and discrete cosine transform (DCT) are widely used in practical application, for instance, the image compression standards JPEG2000. As a classical and effective tool, transform models have been increasingly used in image denoising. Inspired by dictionary learning, Saiprasad et.al [19] proposed a learning sparsifying transform (LST) model. In [19], for any noisy image **X** ∈ *R*^*h*×*l*^, it is first reformed to another resolution as **X**′ ∈ *R*^*p*×*N*^, where each column represents a square patch of the original **X** extracted by a sliding window. Second, a transform matrix **W** ∈ *R*^*p*×*p*^ is randomly initialized to formulate the transform sparse coding problem as follows:

(3) $$\begin{array}{c} \begin{array}{cc} \min & {\left\|WX^{\prime }-\mu \right\|}_{F}^{2}-\lambda \mathrm{lg}\det\left|W\right|,\\ \text{s.t.} & {\left\|\mu_{i}\right\|}_{0}\leq s\forall i, \end{array} \end{array}$$

where ***μ*** ∈ *R*^*p*×*N*^ is the sparse coefficient, ***μ***_*i*_is the column of ***μ***, and *s* is a constant representing the sparse magnitude. The additional regular term *λ*lgdet|**W**| is used to avoid a trivial solution. *λ* is a balance coefficient, and lgdet|**W**| is the log-determinant of **W** with base 10. Ravishankar and Bresler [19] solved the proposed problem by alternately updating **W** and ***μ*** and proved the convergence. To carry forward their achievements, they further proposed a learning doubly sparse transforms (LDST) for IDN [21]. Specifically, **W**′=**B**Φ is adopted to replace the original **W**, where **B** and Φ are both square matrices with the same size. **B** is a transform constrained to be sparse, and Φ is an analysis transform with an efficient implementation. They use the doubly sparse transform model in image denoising and get faster and better results than unstructured transforms. And then, Wen et al. [18, 20] proposed a structured overcomplete sparsifying transform learning (SOSTL) model. The main feature different from aforementioned transform models is that Wen et al. cluster image patches and learns diverse **W** for corresponding patch groups. This process can be formulated as following:

(4) $$\begin{array}{c} \begin{array}{cc} \min & \sum_{k=1}^{K}\left\{\left.\sum_{i\in C_{k}}{\left\|W_{k}X_{i}^{\prime }-\mu_{i}\right\|}_{2}^{2}+\lambda_{k}Q\left(W_{k}\right)\right\},\right.\\ \text{s.t.} & {\left\|\mu_{i}\right\|}_{0}\leq s\forall i,\;\left\{C_{k}\right\}\in G, \end{array} \end{array}$$

where *Q*(**W**)=−log|det**W**|+‖**W**‖_*F*_^2^ is a regular term to prevent trivial solutions. {*Ck*} indicates the specific class of image **X**′, *K* is the number of categories, and *G* is the set of all classes.

### 2.2. Image Domain for IDN

While the transform learning models have achieved great success, in image domain, there also have been proposed various algorithms for IDN. As mentioned before, in the general image denoising model, *F*(**X**) is an additional regularization. The widely studied regularizations include *l*1, *l*2, and *l*1/2 norm, nuclear norm, low-rank property, and so on [22–24]. Focusing on patch form instead of vector form, low-rank property has been attracting a significant research interest. As a convex relaxation of low-rank matrix factorization problem (LRFM), the nuclear norm minimization (NNM) has engrossed more attention [4, 6, 24, 25]. The nuclear norm of an image **X** is defined as ‖**X**‖_*∗*_=∑_*i*_|*σ*_*i*_(**X**)|_1_, where *σ*_*i*_(**X**) is the *i*^th^ singular value of **X**. However, many researchers hold that the minimization of different singular values should be separated. Liu et.al [4] proposed weight nuclear norm minimization (WNNM) for image denoising problems. The weight nuclear norm is defined as ‖**X**‖_*w*,*∗*_=∑_*i*_|*w*_*i*_*σ*_*i*_(**X**)|_1_, and *w* = [*w*_1_, *w*_2_,…, *w*_*n*_] is nonnegative. At this point, we can treat *F*(**X**) as *F*(**X**)=‖**X**‖_*w*,*∗*_, and the denoising model is

(5) $$\begin{array}{c} \underset{X}{\min}{\left\|X-Y\right\|}_{F}^{2}+F\left(X\right),\;F\left(X\right)={\left\|X\right\|}_{w,*}, \end{array}$$

By taking consideration of different singular values, as well as image structure, the WNNM shows strong denoising capability. Meanwhile, Hu et al. [6] proposed truncated nuclear norm regularization (TNNR) for matrix completion. They deemed that the minimization of the smallest min(*m*, *n*)-*r* singular values can maintain the original matrix rank by holding the first *r* nonzero singular values fixed. Using *F*(**X**)=∑_*i*=*r*+1_^min(*m*, *n*)^*σ*_*i*_(**X**), the TNNR constrained model can be written as follows:

(6) $$\begin{array}{c} \underset{X}{\min}{\left\|X-Y\right\|}_{F}^{2}+F\left(X\right),\;F\left(X\right)=\sum_{i=r+1}^{\min\left(m,n\right)}\sigma_{i}\left(X\right). \end{array}$$

TNNR gets a better approximation to the rank function than nuclear norm-based-approaches. Inspired by both WNNM and TNNR, Liu et al. [26] improved the previous algorithms by reweighting the residual error separately and minimizing the truncated nuclear norm of error matrix simultaneously (TNNR-WRE). In their work, *F*(***X***) is considered as follows:

(7) $$\begin{array}{c} F\left(X\right)={\left\|X-Y\right\|}_{*}-tr\left(U_{r}HV_{r}^{\prime }\right), \end{array}$$

where **H** = **X**–**Y**, **U** and **V** are the left and right matrices of **H**'s singular value decomposition (SVD), respectively, and *r* is the truncation parameter. TNNR-WRE further achieves higher accuracy than TNNR.

From above, the nuclear norm-based algorithms usually can get considerable results because of the essential low-rank property in image domain. For taking both advantages of transform domain and image domain in IDN, the sparsifying transform learning and weighted singular values minimization (STLWSM) method is proposed. In contrast to LST, LDST, SOLST, WNNM, TNNR, and TNNR-WRE, the proposed STLWSM jointly takes consideration of sparsity in transform domain and low-rank in image domain. The main results of our work can be enumerated as follows:

- We propose a general framework of image process in both transform domain and image domain, which combines the sparsifying transform learning of image patches and the low-rank property of the original image.
- As image patches can take advantage of the nonlocal similarity existing inherently in the image, we learn the sparsifying transform for each group of similar patches by Euclidean distance.
- For solving the proposed NP-hard problem, we present an efficient alternative optimization algorithm. In practical applications, our method requires limited number of iterations, mostly less than 3, for the final solution.
- We applied our model to IDN, and the results show that STLWSM can achieve evident PSNR (peak signal to noise ratio) improvements over other state-of-the-art methods.

## 3. Proposed Method

In this section, we propose a general framework in both transform domain and image domain. To be clear, we take sparsifying transform learning in transform domain and weighted singular values minimization in image domain simultaneously. To solve this NP-hard problem, an efficient solution is also derived.

### 3.1. Sparsifying Transform Learning and Weighted Singular Values Minimization (STLWSM)

In light of the observations mentioned above, we first introduce a sparsifying learning transform based on image patches and utilize the weighted singular values minimization to improve the image quality.

Given a noisy image **X** ∈ *R*^*h*×*l*^, nonlocal similarity is a well-known patch-based prior, which means that one patch in one image has many similar patches [7–9]. Accordingly, overlapped image patches can be extracted with a sliding window in a fixed step size. For each specific patch, we choose the most similar *M* patches by Euclidean distance [4, 7, 18–20] for potential low-rank structure, and a matrix of **X**_*i*_′ ∈ *R*^*p*×*M*^ is constructed. The patch's size is $\sqrt{p}\times \sqrt{p}$, and the total number *N*′ of **X**_*i*_′depends on the size of the original image **X**, patch size, and step size. After similar patches' aggregation process, in each group, **X**_*i*_′is obtained, and **X**′=[**X**_1_′, **X**_2_′, ..., **X**_*N*′_′] ∈ *R*^*p*×*M*×*N*′^. Following the idea of the transform learning algorithm [18–20], with the obtained **X**_*i*_′ and some initialized ***W***_*i*_, our preliminary model can be formulated as the following:

(8) $$\begin{array}{c} \begin{array}{cc} \underset{W_{i}}{\min} & \sum_{i=1}^{N^{\prime }}{\left\|W_{i}X_{i}^{\prime }-\mu_{i}\right\|}_{F}^{2}-\lambda_{i}Q\left(W_{i}\right),\\ \text{s.t.} & {\left\|\mu_{i}\right\|}_{0}\leq s\forall i. \end{array} \end{array}$$

The definition of *Q*(**W**_*i*_) is the same as one in problem (4), but ***μ***_*i*_ ∈ *R*^*p∗M*^ is the sparse representation of **X**_*i*_′ in transform domain, which is a matrix. Suppose the transform ***W ****i* and sparse coefficient ***μ***_*i*_ have been updated. The denoised patch can be obtained by **X**_*i*_^″^=**W**_*i*_^†^***μ***_*i*_. Obviously, **X**_*i*_^″^ also has low-rank structure; hence, we utilize weighted singular values minimization to approximate the matrix. The unified denoising minimization is

(9) $$\begin{array}{c} \underset{W_{i},\mu_{i}}{\min}\sum_{i=1}^{N^{\prime }}{\left\|W_{i}X_{i}^{\prime }-\mu_{i}\right\|}_{F}^{2}+\alpha_{i}{\left\|\mu_{i}\right\|}_{0}+\beta_{i}{\left\|W_{i}^{†}\mu_{i}\right\|}_{w,*}-\lambda_{i}Q\left(W_{i}\right), \end{array}$$

where *α*_*i*_ and *β*_*i*_are the regularization parameters and usually set empirically. This formulation can minimize the residual in transform domain and the rank of the recovered matrix **X**_*i*_^″^ simultaneously.

### 3.2. Efficient Optimization of the Proposed Model

In this subsection, we introduce an efficient solution for the nonconvex sparsifying transform learning and weighted singular values minimization problem. According to [16–19], the transform learning process is not sensitive to the initialization of ***W***. As a result, with given ***W***, the subproblem of ***μ***_*i*_ can be obtained using cheap hard-thresholding, ${\hat{\mu }}_{i}=Th_{s}\left(\mu_{i}\right)$. Here, *Th*_*s*_(·) is the hard-thresholding operator. And the subproblem of **W**_*i*_ is as follows:

(10) $$\begin{array}{c} \underset{\mu_{i}}{\min}\sum_{i=1}^{N^{\prime }}{\left\|W_{i}X_{i}^{\prime }-\mu_{i}\right\|}_{F}^{2}+\beta_{i}{\left\|W_{i}^{†}\mu_{i}\right\|}_{w,*}-\lambda_{i}\log\left|\det W_{i}\right|+\lambda_{i}{\left\|W_{i}\right\|}_{F}^{2},\\ =\min tr\left\{W_{i}\left(X_{i}^{\prime }{X_{i}^{\prime }}^{T}+\lambda_{i}I_{p}\right)W_{i}^{T}-2W_{i}X^{\prime }\mu_{i}^{T}+\mu_{i}\mu_{i}^{T}\right\}-\lambda_{i}\log\left|\det W_{i}\right|+\min\beta_{i}{\left\|W_{i}^{†}\mu_{i}\right\|}_{w,*}. \end{array}$$

Because the term *β*_*i*_‖**W**_*i*_^†^***μ***_*i*_‖_*w*,*∗*_ is more like a postfix operator, we divide the updating process of **W**_*i*_ into two parts:

(11) $$\begin{array}{c} \left\{\begin{array}{c} \left(a\right)\min tr\left\{W_{i}\left(X_{i}^{\prime }{X_{i}^{\prime }}^{T}+\lambda_{i}I_{p}\right)W_{i}^{T}-2W_{i}X^{\prime }\mu_{i}^{T}+\mu_{i}\mu_{i}^{T}\right\}-\lambda_{i}\log\left|\det W_{i}\right|,\\ \left(b\right)\underset{\mu_{i}}{\min}\sum_{i=1}^{N^{\prime }}{\left\|W_{i}X_{i}^{\prime }-\mu_{i}\right\|}_{F}^{2}+\min\beta_{i}{\left\|W_{i}^{†}\mu_{i}\right\|}_{w,*}. \end{array}\right. \end{array}$$

- The first formula is

(12) $$\begin{array}{c} \min tr\left\{W_{i}\left(X_{i}^{\prime }{X_{i}^{\prime }}^{T}+\lambda_{i}I_{p}\right)W_{i}^{T}-2W_{i}X^{\prime }\mu_{i}^{T}+\mu_{i}\mu_{i}^{T}\right\}-\lambda_{i}\log\left|\det W_{i}\right|. \end{array}$$

Decomposing **X**_*i*_′**X**_*i*_′^*T*^+*λ*_*i*_**I**_*p*_ as **Z**_*i*_**Z**_*i*_^*T*^, **O**_*i*_=**W**_*i*_**Z**_*i*_. Then, **W**_*i*_**X**′***μ***_*i*_^*T*^ can be written as **O**_*i*_**Z**^−1^**X**′***μ***_*i*_^*T*^. Let **O**_*i*_ and **Z**^−1^**X**′***μ***_*i*_^*T*^ have full SVD of **U**Φ**V**^*T*^ and PΨ**Q**^*T*^, respectively. If we take consideration of their diagonal matrix only, the foregoing formula can be rewritten as

(13) $$\begin{array}{c} \min tr\left\{W_{i}\left(X_{i}^{\prime }{X_{i}^{\prime }}^{T}+\lambda_{i}I_{p}\right)W_{i}^{T}-2W_{i}X^{\prime }\mu_{i}^{T}+\mu_{i}\mu_{i}^{T}\right\}-\lambda_{i}\log\left|\det W_{i}\right|,\\ =\min tr\left(O_{i}O_{i}^{T}\right)-2tr\left(O_{i}Z^{-1}X^{\prime }\mu_{i}^{T}\right)-\lambda_{i}\log\left|\det OZ_{i}^{-1}\right|,\\ =\min tr\left(O_{i}O_{i}^{T}\right)-2tr\left(O_{i}Z^{-1}X^{\prime }\mu_{i}^{T}\right)-\lambda_{i}\left(\log\left|\det O_{i}\right|-\log\left|\det Z^{-1}\right|\right),\\ =\min\left[tr\left(\varphi^{2}\right)-2\max\left(tr\left(U_{i}\Phi_{i}V_{i}^{T}\cdot P_{i}\Psi_{i}Q_{i}^{T}\right)\right)-\lambda_{i}\left(\log\left|\det O_{i}\right|\right)\right],\\ \leq \min\sum_{i=1}^{n}\varphi -2\sum_{i=1}^{n}\varphi_{i}^{2}\psi_{i}-\lambda_{i}\sum_{i=1}^{n}\log\varphi_{i}, \end{array}$$

where log|det**Z**^−1^| is the constant and can be omitted. The revised problem is convex for *φ*_*i*_, so the optimizing solution can be found by taking partial differential with respect to *φ*_*i*_ and setting the derivative to 0.

(14) $$\begin{array}{c} 0=\frac{\partial \left(\sum_{i=1}^{n}\varphi_{i}^{2}-\sum_{i=1}^{n}\varphi_{i}\psi_{i}-\lambda_{i}\sum_{i=1}^{n}\log\varphi_{i}\right)}{\partial \varphi_{i}},\\ =\frac{\partial \left(\varphi_{i}^{2}-2\varphi_{i}\psi_{i}-\lambda_{i}\log\varphi_{i}\right)}{\partial \varphi_{i}},\\ =2\varphi_{i}-2\psi_{i}-\lambda_{i}\frac{1}{\varphi_{i}\ln10}. \end{array}$$

Therefore, excluding the nonpositive results, the solution is

(15) $$\begin{array}{c} \varphi_{i}=\frac{\psi_{i}+\sqrt{\psi_{i}^{2}+\left(2\lambda_{i}/\ln10\right)}}{2}. \end{array}$$

To sum up, the transform update step can be computed as follows:

(16) $$\begin{array}{c} {\hat{W}}_{i}={\hat{O}}_{i}Z^{-1}=U_{i}{\hat{\Phi }}_{i}V_{i}^{T}Z_{i}^{-1},\\ =\frac{U_{i}}{2}\left(\Psi_{i}+{\left(\frac{\Psi_{i}+2\lambda_{i}I_{p}}{\ln10}\right)}^{\left(1/2\right)}\right)V_{i}^{T}Z_{i}^{-1}. \end{array}$$

(b) The Second Formula is

(17) $$\begin{array}{c} \underset{\mu_{i}}{\min}\sum_{i=1}^{N^{\prime }}{\left\|W_{i}X_{i}^{\prime }-\mu_{i}\right\|}_{F}^{2}+\beta_{i}{\left\|W_{i}^{†}\mu_{i}\right\|}_{w,*}. \end{array}$$

With fixed ${\hat{W}}_{i}$ obtained in step (a), this part can be simply seen as

(18) $$\begin{array}{c} \underset{\mu_{i}}{\min}\sum_{i=1}^{N^{\prime }}{\left\|W_{i}X_{i}^{\prime }-\mu_{i}\right\|}_{F}^{2}+\beta_{i}\cdot L_{W_{i}^{†}\mu_{i}}w_{i}\sum_{W_{i}^{†}\mu_{i}}R_{W_{i}^{†}\mu_{i}}, \end{array}$$

where **L**_**W**_*i*_^†^***μ***_*i*__*w*_*i*_Σ_**W**_*i*_^†^***μ***_*i*__**R**_**W**_*i*_^†^***μ***_*i*__=SVD(**W**_*i*_^†^***μ***_*i*_), and **W**_*i*_^†^***μ***_*i*_ represents the denoised matrix. Following Liu et.al [4], a desirable weighting vector ***W***_*i*_ in image domain can be given as

(19) $$\begin{array}{c} w_{i}=c\frac{\sqrt{M}}{\left(\sigma_{i}\left(W_{i}^{†}\mu_{i}\right)+\varepsilon \right)}, \end{array}$$

where *σ*_*i*_(**W**_*i*_^†^***μ***_*i*_)is the *i*^th^ singular of **W**_*i*_^†^***μ***_*i*_, *c* is a positive constant, and *ε* = 10^−16^ is to avoid dividing by zero. And the second formula's optimal solution is

(20) $$\begin{array}{c} {\hat{X}}_{i}^{²}=L_{X}S_{w}\left(\sum_{X_{i}^{²}}\right)R_{X_{i}^{²}}^{T}, \end{array}$$

where **X**_*i*_^″^**=W**_*i*_^†^***μ***_*i*_ and the soft-thresholding operator **S**_*w*_(**∑**_*i*_) is defined as **S**_*w*_(**∑**_*i*_)=max(**∑**_*i*_ − *w*_*i*_, 0).

The summary of our optimization solution is presented in Algorithm 1 where the similar patches are determined by Euclidean distance.

## 4. Experiment Results

**Figure 1:**
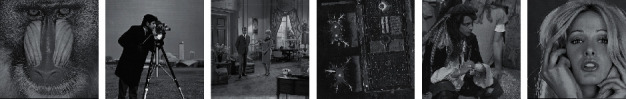
Original gray images.

**Figure 2:**
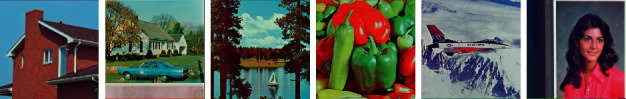
Original color images.

**Figure 3:**
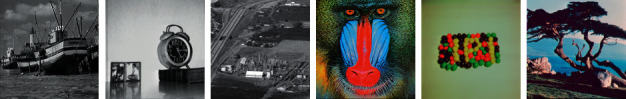
Original images of size 512*∗*512.

In this section, we choose 25, 12, 15, and 10 reference images with a size of 256*∗*256 from TID2008 [27], USC-SIPI1, Live-IQAD [28], and IVC-SQDB [29] to test the image denoising effects, respectively. As we use six different noise levels to the test images in our experiments, the total number of distorted images is 372. Some representative images from USC-SIPI database are shown in Figures 1 and 2. Four recently proposed methods, including the patch-based algorithm GSR, weighted nuclear norm WNNM, sparsity learning transform scheme SOLST and sparsity transform learning, and the low-rank model STROLLR, are adopted as contrasts. The noisy images are obtained by additional Gaussian noise with *σ*_*n*_ = 15, 20, 30, 40, 50, and 75. All competing algorithms use their default settings, which has been finely tuned and deeply verified in their original publications. Since our method is derived from both the schemes of image domain and transform domain, we set our parameters the same as the representative methods in these two domains, i.e., WNNM and SOLST, for fairness. That is, for the image denoising application, when*σ*_*n*_ ≤ 20, p is 6, M is 70, and *λ*_*i*_is 0.54. When 20 < *σ*_*n*_ ≤ 40, p is 7, M is 90, and *λ*_*i*_is 0.56. When 40 < *σ*_*n*_ ≤ 60, p is 8, M is 120, and *λ*_*i*_is 0.58. And when*σ*_*n*_is set others, p is 9, M is 140, and *λ*_*i*_is 0.58. In addition, 6 images of 512*∗*512 from USC-SIPI (Figure 3) are used in image inpainting application. For the image inpainting application, we also follow the similar setting rule. The balance parameters *α*_*i*_ and *β*_*i*_are both set as *α*_*i*_=*β*_*i*_=10*∗*‖**X**_*i*_′‖_*F*_^2^.  Table 1 shows the detailed parameter setting in our experiments where the texts in bracket are used for the 512*∗*512 images, while the plain ones are for the 256*∗*256 images.

The peak signal-to-noise ratio (PSNR) and structural similarity index measure (SSIM) are used to evaluate the quality of the denoised images. PSNR is defined by

(21) $$\begin{array}{c} \text{PSNR}=10*\log_{10}\left(\frac{255}{\text{MSE}}\right), \end{array}$$

where MSE is the mean squared error between the original image and the denoised one. SSIM is defined as [28, 30]

(22) $$\begin{array}{c} \text{SSIM}\left(x,y\right)=\frac{\left(2\mu_{x}\mu_{y}+C_{1}\right)\left(2\sigma_{xy}+C_{2}\right)}{\left(\mu_{x}^{2}+\mu_{y}^{2}+C_{1}\right)\left(\sigma_{x}^{2}+\sigma_{y}^{2}+C_{2}\right)}, \end{array}$$

where *x* and *y* represent the original image and the denoised one, respectively, ***μ***_*x*_and ***μ***_*y*_are the mean values of *x* and *y*, *σ*_*x*_ and *σ*_*y*_are the variances, and *σ*_*xy*_is the covariance. *C*1 and *C*2 denote two stabilization variables.

For a thorough comparison, we list the average denoising results from all the 372 distorted images in Table 2. Also, the experimental results from all the gray images of USC-SIPI are provided in Table 3.

From these two tables, we can observe that among the competing algorithms, GSR also adopts the nonlocal similarity that groups image patches for low-rank structure. However, it requires too much iterations in practical applications, e.g., 100 or even up to 200 times. In contrast, WNNM needs fewer iterations, around 14, and achieves pretty good results than other 3 algorithms at an average of 8.26 dB for gray images. In the meantime, the proposed STLWSM needs the least iterations and achieves best performance.

**Figure 4:**
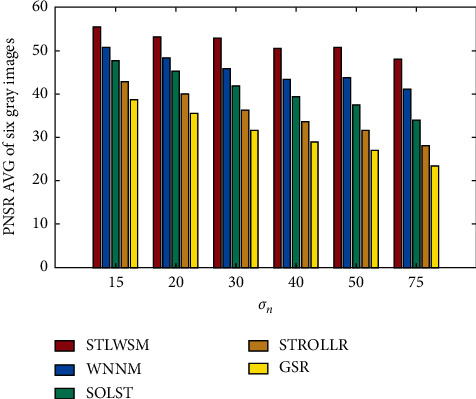
PSNR AVG of gray images denoising results.

SOLST and STROLLR are both transform algorithms and have hard-to-catch efficiency. STROLLR trains transform matrices for each group, while SOLST combines nonlocal low-rank and transform learning, and they also achieved better results than STROLLR at an average of 5.54 dB. In Table 3, the numerical results of the proposed STLWSM are all made bold that means the best one among the five algorithms. It is evident that the proposed method has achieved visible improvement in PSNR under all kinds of noise levels at an average of 13.61 dB. More visual results are shown in Figure 4, in which our method clearly outperforms all other methods.

**Figure 5:**
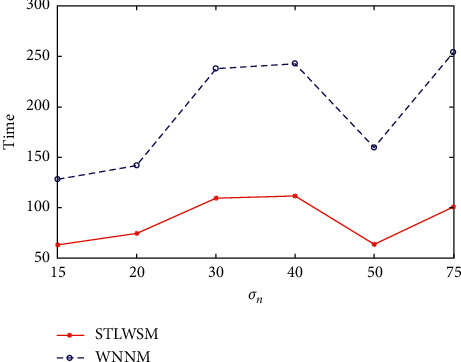
Elapsed time comparison in gray images.

Moreover, considering that GSR needs too much iterations, and pure transform learning algorithms are extremely faster; we compare our time consummation against WNNM, and the results are shown in Figure 5. It can be seen that our method spends much less time than WNNM, at an average of 55.46%.

**Figure 6:**
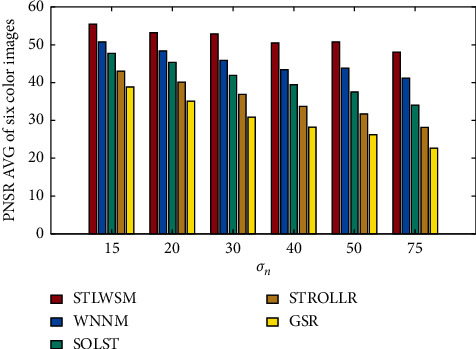
PSNR AVG of color images denoising results.

**Figure 7:**
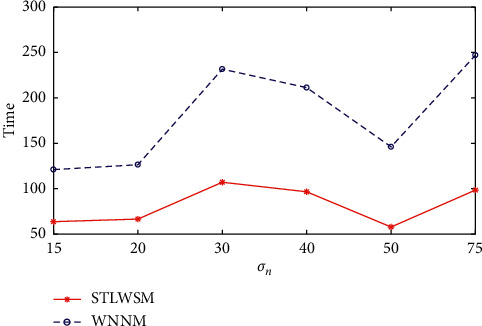
Elapsed time comparison in color images.

**Figure 8:**
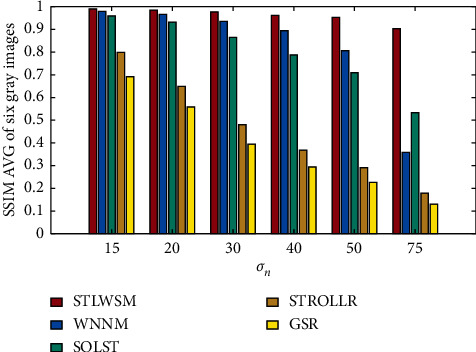
SSIM AVG of gray images denoising results.

**Figure 9:**
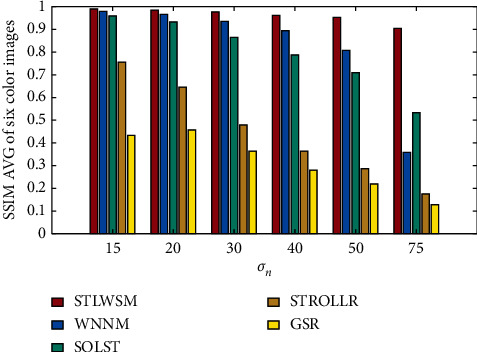
SSIM AVG of color images denoising results.

Our algorithm also has good scalability; we further use RGB images in IDN, and experiments results show that the proposed STLWSM still outperform than other algorithms, and specific numerical comparisons are shown in Table 4. Again, Figures 6 and 7, respectively, show the visual results in terms of the average PSNR and the elapsed time, which also demonstrate our superiority against other competitors. Figures 8 and 9 show the visual results of average SSIM comparison of gray images and color images, respectively. It can be seen that our method can hold denoised image structure even with high noise rate.

**Figure 10:**
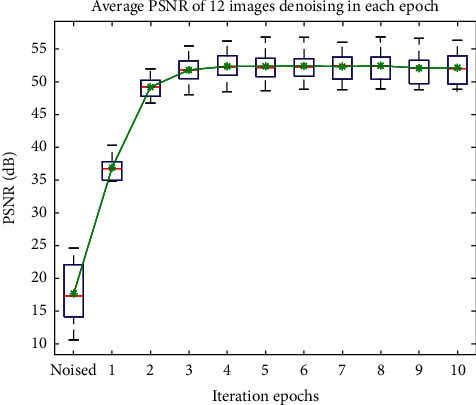
Average PSNR of 12 images denoising in each epoch of different image noise levels.

For detailed display of the efficiency of our algorithm, we provide its generated results versus different iterations (up to 10). The experimental results are shown in Figure 10. All 12 images' PSNR values are averaged for each noise level. The PSNR value of the original noisy images in different noise levels is shown as the starting point where the top black line is the max value of 24.63, the bottom black line is the min value of 10.65, and the red line represents the median of 17.64. And the green star is an average of 17.72.  Figure 8 shows that our algorithm has a fast constringency speed and needs limited number of iterations, mostly 3, for the final solution.

We also applied our method in image inpainting with 6 images in sizes of 512*∗*512, and the degenerated images are obtained by multiplying with a random logical matrix in an element-wise manner, and the missing rates are set as *σ*_*m*_ = {15%, 20%, 30%, 40%, 50%}. The image inpainting results are shown in Table 5. The original images are shown in Figure 3. The results show that all methods achieve admirable inpainting results for filling in missing pixels, and the proposed STLWSM still outperforms all the other state-of-the-art algorithms. Taking into account the image denoising results, our STLWSM has better robustness with much less PSNR changes compared to other competing approaches.

## 5. Conclusions

In this paper, we have proposed a unified framework of image denoising using both knowledge from image domain and transform domain, namely sparsifying transform learning and weighted singular values minimization (STLWSM). Specifically, we learned the transform matrix for each group of patches with similar structure. After obtaining the optimized transform matrix and the sparse coefficient with an efficient optimization algorithm, we further restored the image patch groups through their low-rank prior. By adopting STLWSM to all the groups, a denoised image can be reconstructed. For both gray images and color images, experimental results show that, the proposed model can achieve visible improvement in PSNR over other state-of-the-art approaches. Our efficient optimization algorithm also costs much less running time compared to the typical image domain-based method. Note that while the pure transform learning methods run faster than STLWSM, they perform poorer with a large margin. To further improve, the efficiency of our framework will be our main work in the near future.

## Figures and Tables

**Algorithm 1:**
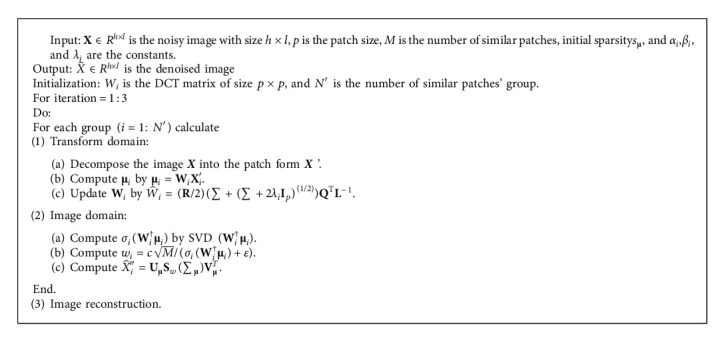
Efficient Solution of STLWSM.

**Table 1:** Parameter setting in our experiments.

*σ*_*n*_(*σ*_*m*_)	15 (15%)	20 (20%)	30 (30%)	40 (40%)	50 (50%)	75
*P*	6 (12)		7 (14)		8 (16)	9
*M*	70 (200)		90 (260)		120 (300)	140
*λ*_*i*_	0.54 (0.54)		0.56 (0.56)		0.58 (0.58)	0.58
*α*_*i*_			10^*∗*^‖**X**_*i*_′‖_*F*_^2^(10^*∗*^‖**X**_*i*_′‖_*F*_^2^)		
*β*_*i*_			10^*∗*^‖**X**_*i*_′‖_*F*_^2^(10^*∗*^‖**X**_*i*_′‖_*F*_^2^)		

**Table 2:** Average denoising results with different noise level (PSNR/SSIM).

*σ*_*n*_	GSR	WNNM	SOLST	STROLLR	STLWSM
15	38.63/0.574	50.78/0.935	47.74/0.914	42.99/0.677	55.49/0.983
20	35.25/0.431	48.36/0.925	45.34/0.894	40.11/0.584	53.20/0.978
30	31.23/0.293	45.88/0.903	41.91/0.822	36.67/0.465	52.90/0.973
40	28.59/0.216	43.40/0.874	39.45/0.790	33.72/0.375	50.52/0.962
50	26.60/0.165	43.82/0.811	37.54/0.734	31.70/0.328	50.75/0.955
75	23.04/0.095	41.19/0.488	34.05/0.609	28.17/0.254	48.07/0.920

**Table 3:** Gray images' denoising results (PSNR/SSIM).

Image	*σ*_*n*_	GSR	WNNM	SOLST	STROLLR	STLWSM
Baboon	15	38.27/0.765	50.89/0.981	47.76/0.960	43.00/0.752	55.74/0.992
	20	35.41/0.650	48.42/0.967	45.35/0.933	40.12/0.640	53.36/0.987
	30	31.60/0.478	45.98/0.937	41.91/0.865	36.34/0.469	53.08/0.979
	40	29.01/0.359	43.45/0.896	39.45/0.788	33.70/0.356	50.63/0.963
	50	27.03/0.275	43.89/0.809	37.54/0.710	31.71/0.280	50.88/0.955
	75	23.47/0.154	41.21/0.360	34.05/0.535	28.18/0.170	48.15/0.906

Camera	15	39.32/0.577	50.74/0.979	47.72/0.959	42.89/0.741	55.32/0.990
	20	35.77/0.429	48.36/0.964	45.32/0.932	40.04/0.629	53.11/0.985
	30	31.67/0.295	45.89/0.935	41.92/0.864	36.31/0.458	52.81/0.976
	40	29.03/0.225	43.43/0.894	39.45/0.788	33.69/0.347	50.47/0.961
	50	27.04/0.179	43.79/0.806	37.54/0.709	31.70/0.271	50.71/0.952
	75	23.47/0.112	41.16/0.359	34.05/0.535	28.17/0.162	48.06/0.902

Couple	15	38.82/0.719	50.82/0.980	47.75/0.960	42.95/0.746	55.57/0.991
	20	35.61/0.584	48.40/0.967	45.34/0.933	40.09/0.634	53.26/0.986
	30	31.64/0.411	45.87/0.936	41.90/0.865	36.33/0.463	52.98/0.978
	40	29.02/0.305	43.44/0.895	39.45/0.288	33.76/0.350	50.57/0.963
	50	27.03/0.233	43.82/0.807	37.54/0.711	31.69/0.275	50.83/0.954
	75	23.47/0.132	41.19/0.359	34.05/0.535	28.18/0.166	48.14/0.905

Lax	15	38.39/0.751	50.80/0.980	47.76/0.959	42.64/0.717	55.65/0.992
	20	35.46/0.636	48.38/0.966	45.34/0.931	39.86/0.600	53.29/0.986
	30	31.61/0.470	45.88/0.935	41.91/0.863	36.20/0.425	52.97/0.977
	40	29.01/0.357	43.39/0.894	39.45/0.787	33.59/0.313	50.55/0.962
	50	27.02/0.277	43.83/0.806	37.53/0.710	31.61/0.241	50.78/0.953
	75	23.47/0.160	41.20/0.359	34.05/0.536	28.11/0.140	48.08/0.903

Man	15	38.79/0.690	50.74/0.979	47.73/0.959	42.88/0.739	55.37/0.990
	20	35.61/0.552	48.36/0.965	45.33/0.931	40.03/0.627	53.13/0.985
	30	31.64/0.381	45.89/0.935	41.90/0.863	36.29/0.454	52.85/0.976
	40	29.02/0.279	43.38/0.894	39.45/0.786	33.67/0.342	50.48/0.961
	50	27.03/0.212	43.77/0.806	37.53/0.708	31.67/0.267	50.71/0.952
	75	23.47/0.119	41.19/0.358	34.05/0.533	28.15/0.159	48.05/0.903

Woman1	15	39.02/0.648	50.81/0.996	47.75/0.960	43.08/0.759	55.53/0.991
	20	35.68/0.499	48.34/0.990	45.34/0.933	40.18/0.650	53.22/0.986
	30	31.66/0.333	45.87/0.936	41.90/0.865	36.39/0.479	52.91/0.978
	40	29.02/0.240	43.38/0.895	39.45/0.788	33.73/0.366	50.52/0.962
	50	27.03/0.181	43.81/0.807	37.54/0.710	31.71/0.289	50.74/0.954
	75	23.47/0.102	41.19/0.358	34.05/0.534	28.20/0.177	48.06/0.904

**Table 4:** Color images' denoising results (PSNR/SSIM).

Image	*σ*_*n*_	GSR	WNNM	SOLST	STROLLR	STLWSM
House	15	38.89/0.507	50.87/0.980	47.76/0.960	43.06/0.756	55.73/0.992
	20	35.08/0.550	48.43/0.967	45.35/0.933	40.14/0.645	53.35/0.987
	30	30.90/0.448	45.96/0.937	41.91/0.865	36.37/0.477	53.04/0.978
	40	28.24/0.356	43.40/0.896	39.45/0.788	33.73/0.364	50.63/0.963
	50	26.24/0.268	43.86/0.808	37.54/0.710	31.71/0.287	50.86/0.955
	75	22.67/0.152	41.19/0.359	34.05/0.534	28.19/0.176	48.14/0.905

House 2	15	38.20/0.329	50.78/0.980	47.74/0.960	43.19/0.770	55.55/0.992
	20	34.87/0.319	48.37/0.967	45.33/0.933	40.26/0.663	53.25/0.986
	30	30.85/0.265	45.88/0.937	41.90/0.865	36.44/0.466	52.97/0.978
	40	28.22/0.211	43.42/0.896	39.44/0.789	33.80/0.383	50.57/0.963
	50	26.23/0.172	43.82/0.809	37.54/0.710	31.76/0.278	50.84/0.955
	75	22.67/0.109	41.22/0.358	34.05/0.533	28.21/0.190	48.15/0.907

Lake	15	38.10/0.484	50.67/0.979	47.71/0.959	42.93/0.746	55.29/0.990
	20	34.83/0.461	48.27/0.965	45.31/0.932	40.08/0.635	53.09/0.985
	30	30.84/0.381	45.83/0.935	41.89/0.864	36.32/0.466	52.82/0.977
	40	28.22/0.291	43.38/0.895	39.44/0.788	33.71/0.354	50.48/0.962
	50	26.23/0.226	43.78/0.808	37.54/0.710	31.68/0.278	50.72/0.954
	75	22.67/0.130	41.22/0.359	34.04/0.534	28.16/0.169	48.08/0.906

Pepper	15	38.52/0.535	50.74/0.978	47.74/0.959	42.94/0.744	55.28/0.989
	20	34.97/0.492	48.33/0.964	45.33/0.932	40.07/0.632	53.06/0.984
	30	30.88/0.439	45.84/0.933	41.98/0.864	39.52/0.476	52.73/0.975
	40	28.23/0.344	43.35/0.892	39.45/0.787	33.69/0.348	50.45/0.959
	50	26.24/0.279	43.81/0.805	37.54/0.709	31.70/0.272	50.61/0.950
	75	22.67/0.158	41.18/0.358	34.05/0.534	28.16/0.164	47.95/0.900

Plane	15	38.44/0.451	50.82/0.980	47.74/0.961	43.31/0.782	55.57/0.992
	20	34.94/0.431	48.37/0.967	45.34/0.939	40.35/0.676	53.25/0.987
	30	30.87/0.348	45.88/0.937	41.91/0.867	36.51/0.511	52.95/0.979
	40	28.23/0.265	43.41/0.896	39.45/0.790	33.85/0.397	50.55/0.963
	50	26.23/0.204	43.85/0.809	37.54/0.712	31.81/0.318	50.79/0.955
	75	22.67/0.117	41.17/0.358	34.05/0.534	28.23/0.200	48.14/0.906

Woman 2	15	38.82/0.398	50.73/0.979	47.74/0,959	42.89/0.737	55.36/0.990
	20	35.07/0.390	48.32/0.965	45.34/0.932	40.03/0.623	53.11/0.985
	30	30.90/0.302	45.86/0.934	41.90/0.863	36.29/0.451	52.73/0.976
	40	28.23/0.227	43.42/0.893	39.44/0.786	33.67/0.338	50.39/0.960
	50	26.24/0.174	43.84/0.805	37.53/0.708	31.67/0.264	50.61/0.951
	75	22.67/0.102	41.14/0.357	34.04/0.533	28.14/0.157	47.93/0.901

**Table 5:** Images inpainting results of size 512*∗*512.

Image	*σ*_*m*_ (%)	WNNM	SOLST	STROLLR	STLWSM
Boats	15	57.88|	56.51	56.88	58.05
	20	57.36	56.19	56.32	57.82
	30	56.57	55.76	55.87	57.32
	40	56.08	55.18	55.53	56.64
	50	55.86	54.79	55.05	55.63

Clock	15	54.39	53.85	53.91	55.12
	20	54.16	53.45	53.62	54.81
	30	53.99	53.76	53.94	55.52
	40	53.42	53.14	53.49	53.79
	50	52.15	52.14	52.50	52.71

Factory	15	59.11	58.18	58.26	59.68
	20	58.85	57.73	57.76	59.45
	30	58.26	56.16	56.35	58.98
	40	57.55	55.14	55.46	57.57
	50	56.86	54.49	55.11	56.66

Baboon	15	57.95	56.18	57.97	58.54
	20	57.25	55.85	56.95	57.94
	30	57.09	55.47	56.12	57.58
	40	56.56	54.95	55.27	57.07
	50	56.01	54.35	54.48	56.18

Beans	15	56.13	54.26	55.18	56.57
	20	55.85	54.19	54.79	56.19
	30	54.32	53.92	54.22	55.55
	40	53.61	53.14	53.29	54.74
	50	52.52	51.95	52.03	53.67

Tree	15	57.15	56.74	56.91	57.85
	20	57.08	56.34	56.66	57.64
	30	56.59	55.73	56.71	57.11
	40	54.73	54.67	54.71	55.26
	50	53.56	53.22	53.34	53.95

## Data Availability

The image data are provided in the manuscript, and all images can be found in http://sipi.usc.edu/database/. The codes of this article are available in https://github.com/Yapan0975/STLWSM.
